# Immuno-thermal ablations – boosting the anticancer immune response

**DOI:** 10.1186/s40425-017-0284-8

**Published:** 2017-10-17

**Authors:** Ryan Slovak, Johannes M. Ludwig, Scott N. Gettinger, Roy S. Herbst, Hyun S. Kim

**Affiliations:** 1 Division of Interventional Radiology, Department of Radiology and Biomedical Imaging, Yale School of Medicine, 330 Cedar Street, New Haven, CT 06510 USA; 2 University of Connecticut School of Medicine, 263 Farmington Avenue, Farmington, CT 06032 USA; 3 Department of Diagnostic and Interventional Radiology and Neuroradiology, University Hospital Essen, University of Duisburg-Essen, Hufelandstr. 55, 45147 Essen, Germany; 4 Division of Medical Oncology, Department of Internal Medicine, Yale School of Medicine, 330 Cedar Street, New Haven, CT 06510 USA; 5 Yale Cancer Center, Yale School of Medicine, New Haven, 330 Cedar Street, New Haven, CT 06510 USA; 6 Yale School of Medicine, Yale Cancer Center, 333 Cedar Street, P.O. Box 208042, New Haven, CT 06520 USA

**Keywords:** Combination Therapy, Cryoablation, Radiofrequency ablation, Microwave ablation, Checkpoint inhibition

## Abstract

The use of immunomodulation to treat malignancies has seen a recent explosion in interest. The therapeutic appeal of these treatments is far reaching, and many new applications continue to evolve. In particular, immune modulating drugs have the potential to enhance the systemic anticancer immune effects induced by locoregional thermal ablation. The immune responses induced by ablation monotherapy are well documented, but independently they tend to be incapable of evoking a robust antitumor response. By adding immunomodulators to traditional ablative techniques, several researchers have sought to amplify the induced immune response and trigger systemic antitumor activity. This paper summarizes the work done in animal models to investigate the immune effects induced by the combination of ablative therapy and immunomodulation. Combination therapy with radiofrequency ablation, cryoablation, and microwave ablation are all reviewed, and special attention has been paid to the addition of checkpoint blockades.

## Background

In clinical routine, therapeutic techniques such as thermal ablation and surgical resection are used to target local malignancies for destruction. Due to the locoregional nature of these very precise interventions, small, distal metastases often escape destruction. Unsurprisingly these micrometastases can be responsible for tumor recurrence after treatment [1].

One potential way to overcome the challenge of micrometastases is to induce systemic antitumor immunity by activating the immune system. Thermal ablative techniques such as cryoablation, radiofrequency ablation (RFA), microwave ablation (MWA), and focused ultrasound (FUS) have all been shown to trigger an immune response [2–5]. By destroying readily accessible tumors, ablative therapies make tumor antigens available as an in-situ cancer vaccine which can lead to the initiation of a systemic antitumor immune response that can affect and potentially eliminate occult, metastatic tumors [3, 4]. The phenomenon whereby a locally applied therapy triggers a distal antitumor response is termed the abscopal effect [6].

The magnitude of the abscopal effect induced by ablative therapy alone has proven to be either weak and insufficient or counterproductive [2, 7]. Recently, interest has shifted towards exploring the potential synergy between ablative techniques and immunotherapies. By combining these two forms of oncologic treatment, investigators aim to overcome immune regulation and enhance long-term, systemic antitumor immunity [2, 4]. Research into these combination therapies is just beginning and while some success has been seen in clinical trials, many studies continue to be conducted in animal models [8–10]. With this review, we will summarize the data gathered from the study of combination ablation immunotherapy in animal models (Table 1).

**Table 1:** A concise summary of significant findings from preclinical studies combining immunotherapy with thermal ablation

Significance At A Glance						
Radiofrequency Ablation	Survival	Rechallenge	Tumor Volume	Cytolytic Activity	CD4+ Tumor Infiltration	CD8+ Tumor Infiltration
+CpG B [31]	↑	NS	–	↑	–	–
+Ex Vivo Stimulated Dendritic Cells [38, 46]	↑	–	↓	↑	↑	↓
+Poxviral CEA/TRICOM Vaccine [40]	NS	–	↓	–	–	–
+CC Chemokine Ligand 3 [41]	–	–	↓	–	↑	↑
+Anti-CTLA-4 Antibodies [10]	–	↑	–	–	–	–
+Anti-CD25 Antibodies [10]	–	↑	–	–	–	`−
+Anti-PD-1 Antibodies [16]	↑	–	↓	–	–	–
Cryoablation	Survival	Rechallenge	MHC I Presentation	MHC II Presentation	CD4+ Tumor Infiltration	CD8+ Tumor Infiltration
+CpG ODN [12]	↑	↑	↑	↑	–	–
+Ex Vivo Immature Dendritic Cells [37]	↑	↑	–	–	–	–
+ TLR7 Agonist Imiquimod [11]	–	↑	–	–	–	–
Anti-CTLA-4 Antibodies [10, 15]	↑	↑	–	–	↑	↑
Anti-CD25 Antibodies [10]	–	↑	–	–	–	–
+Cyclophosphamide [44]	↑	↑	–	–	–	–
Microwave Ablation	Survival	Rechallenge	Tumor Volume	Th1/Th2 Cytokine Ratio	CD4+ Tumor Infiltration	CD8+ Tumor Infiltration
+OK 432 [42]	↑	↑	–	↑	↑	↑
+GM-CSF Microspheres [45]	–	↑	↓	–	–	–
+GM-CSF Microspheres & Anti-CTLA-4 Antibodies [45]	↑	↑	↓	–	–	–

↑ Significantly improved response (*p* < 0.05), ↓ significant decrease (*p* < 0.05), NS not significant

## Immunostimulatory effects induced by ablation

There are a wide variety of ablation techniques designed to destroy solid organ tumors. Many of these methods have been in use for decades, but their ability to trigger systemic immune responses is just beginning to be fully appreciated. Though the goals of ablation are similar to those of surgical resection, ablation differs in that the tumor material is left in situ. Even with the bulk of the tumor destroyed, antigenic remnants persist. This aspect of ablation is responsible for its ability to trigger a systemic antitumor immune response where surgical resection would not [2, 11].

**Fig. 1:**
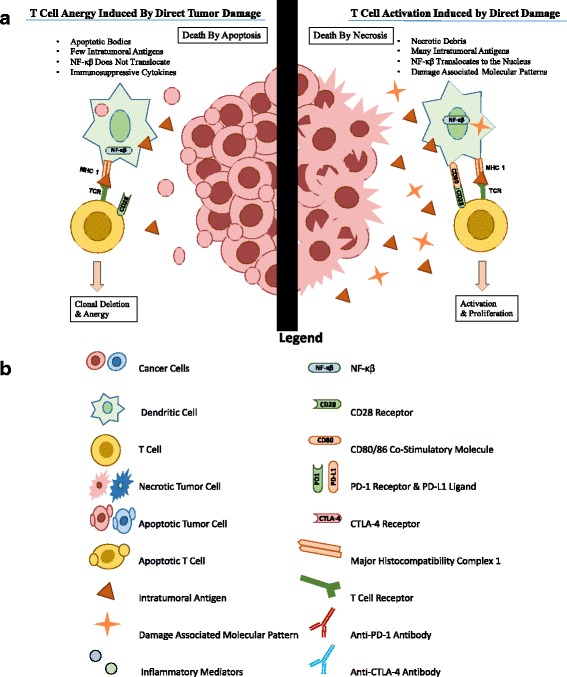
**a** Indirect ablative damage triggers apoptotic cell death and does not induce co-stimulator expression on DCs. In contrast, direct ablative damage releases DAMPs that activate the NF-κβ pathway and induce co-stimulator expression in DCs, thereby promoting the activation and proliferation of T cells [2, 3, 7, 12, 13]. **b** Legend for Figs. 1 and 2

**Fig. 2:**
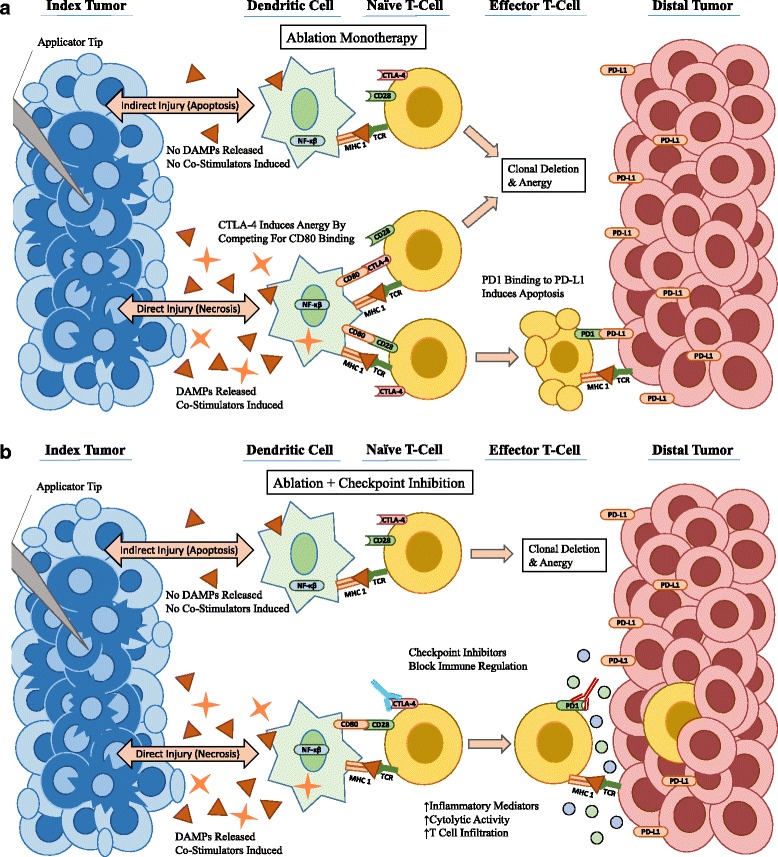
**a** Ablation therapy alone is often sufficient to activate the immune system; however, the CTLA-4 and PD-1 checkpoints regulate and inhibit the induction of a more robust immune response [2, 3, 7, 12, 13]. B - Both anti -PD-1 and anti-CTLA-4 antibodies enhance the immune response induced via ablation monotherapy by blocking regulatory checkpoints. [2, 3, 7, 12–14, 35, 54]

Cryoablation utilizes expanding argon gas to induce a freeze-thaw cycle in targeted lesions that results in necrotic cell death in a small radius around the probe. The cells that die via necrosis release preserved intracellular organelles, antigens, and damage associated molecular patterns (DAMPs) such as DNA and heat shock proteins (HSPs) [7]. Dendritic cells (DCs) that phagocytize these DAMPs activate the nuclear factor kappa-light-chain-enhancer of activated B cells (NF-κβ) pathway, which then promotes the expression of co-stimulatory CD80/86 molecules [12, 13]. Dendritic cells that present antigens on major histocompatibility complex (MHC) molecules and show co-stimulators stimulate T-cells and promote a systemic immune response [2, 14, 15]. Some authors have referred to this ability of cryotherapy to load dendritic cells as producing an “in-vivo dendritic cell vaccine” [16]. The immunostimulatory response induced by cryoablation alone has been noted as the most potent among ablative therapies, as evidenced by significantly higher post-ablative levels of serum interleukin-1 (IL-1), IL-6, NF-κβ, and tumor necrosis factor-α (TNF-α) [2]. Peripheral to the site of cryoablation, sublethal temperatures induce apoptotic cell death [2, 7, 17]. Cells that die by apoptosis also release antigens that can be picked up by dendritic cells, but they do not typically release DAMPs. Without phagocytizing DAMPS, the NF-κβ pathway is not activated and expression of CD80 and CD86 is not induced [12, 13]. Without these co-stimulators, T-cell anergy or even clonal deletion may occur, thereby suppressing the immune response (See Figs. 1 and 2) [3, 14]. The necrotic cell death therefore seems critical for inducing a systemic immune response with cryotherapy. Thus, cryoablation alone can induce both an immunostimulatory and an immunosuppressive effect. Whether stimulation or suppression prevails is dependent on whether there is more necrosis or apoptosis, and the proportion of these responses may vary over time [18]. By combining cryoablation with immunotherapy, many investigators have sought to enhance the dendritic cell loading or inhibit the regulatory response induced by the lack of co-stimulators, thereby improving immunostimulation and limiting immunosuppression.

Radiofrequency ablation induces cell death via direct hyperthermic injury and coagulative necrosis. Just as in cryotherapy, necrotic cell death caused by RFA releases intracellular antigens and DAMPs such as HSPs and high mobility group protein B1 (HMGB1) as well as RNA and DNA that can be picked up by dendritic cells and go on to stimulate a systemic immune response. Outside of the focal zone, diffusion of heat creates a transition zone where sublethal temperatures induce apoptotic cell death. Heat Shock Protein 70 is particularly elevated in this transitional zone, and it is believed that it plays a key role in mediating RFA’s immunostimulatory effect [3]. Hours to days after RFA, levels of IL-1β, IL-6, IL-8, and TNF-α have all been shown to be increased [2]. Radiofrequency ablation alone has also been shown to cause a persistent increase in tumor specific antibodies, CD4+ T cells, CD8+ T Cells and to decrease levels of CD25+ FoxP3+ regulatory T cells [19, 20]. In spite of these results, RF has also been proven to cause hypoxia driven metastatic tumor growth distal to the site of treatment [21, 22]. Researchers are beginning to investigate the ability of additive immunotherapies to enhance the immunostimulatory and diminish the oncogenic effects of RFA.

Microwave ablation utilizes an oscillating electromagnetic field to release kinetic energy as heat that damages nearby cells via direct hyperthermic injury. Compared to cryoablation and RFA, the immune response induced by MWA is relatively paltry. While IL-1, IL-6, and HSP 70 are all elevated following MWA, the magnitude of their induction is significantly less than what follows after cryoablation and RFA [23, 24]. Perhaps due to this comparatively poor immune induction, MWA in combination with immunotherapy has not been studied in animal models as extensively as the other ablative methods.

Focused ultrasound (FUS) is an additional image-guided ablative technique that is available in a range of input energies. High intensity focal ultrasound (HIFU) is similar to the other hyperthermic modalities in that it generates cell death at a focal point via coagulative necrosis and apoptotic death in a transitional zone [3, 4, 25]. Cells destroyed by HIFU release intratumoral antigens including several HSPs and damage associated molecular patterns (DAMPs) that can go on to stimulate innate and adaptive immune responses [4, 25]. Following HIFU, augmented levels of IL-2, IFN-γ, and TNF-α and decreased levels of IL-4, IL-5, and IL-10 have been observed. Additionally, the immunosuppressive factors VEGF, TGF-β1 and TGF-β2 were all shown to be decreased after HIFU [4, 25]. However, just like microwave ablation, the immune response induced by HIFU has been shown to be minimal in comparison to cryoablation and RFA [2]. Some have hypothesized that the reason for this restricted response is that the coagulative necrosis generated by HIFU destroys much the structure and vascularity of tumors, thus limiting the ability of immune cells to reach and interact with the tumor [5]. With this in mind, low energy focused ultrasound (LOFU) was designed to deliver a limited amount of energy that would be immunogenic while also being incapable of inducing coagulative necrosis. LOFU has been shown to reduce the expression of T cell anergy inducing genes, thereby discouraging tumor induced immune tolerance. Additionally, following treatment with LOFU, calreticulin was seen to be redistributed in B16 cells and the expression of HSP70, MHC II, and B7 were all shown to be increased, indicating treatment induced changes in the levels of stress proteins and dendritic maturation states [5]. Unfortunately, very little work has been done to study the benefits of combining immunotherapy with either HIFU or LOFU in animal models.

Yttrium-90 (Y90) radioembolization is used for the treatment of primary liver tumors and liver metastases. This technique allows for the delivery of a β-emitting radioactive isotope directly into target lesions. An immune-mediated abscopal effect has been recorded after radioembolization. The likely mechanism of this effect was described as resulting from β-emission induced immunogenic cell death that caused tumor cells to release chemokines (Monocyte Chemoattractant Protein-1 & CXCL16), cytokines TNF-α, IL-1, & IL-16), and danger signals (ATP, calreticulin, & HMGB1) as well as tumor antigens. Dendritic cells could then pick up and present the tumor antigens to CD4+ and CD8+ T cells resulting in a systemic cell-mediated immune response [26]. The immunogenic effects of radioembolization have yet to be studied in animal models. Several studies have delved into the synergistic effects of combining radiation in the form of either Y90 or stereotactic ablative radiotherapy (SABR/SBRT) with immunomodulation, but they remain outside the scope of this review, as these treatments have not yet been studied in preclinical, animal models [27–29].

Yet another form of thermal ablation available to treat malignant tumors is photothermal ablation. This relatively new technique involves selective deposition of nanoparticles which can then be targeted with lasers to trigger hyperthermia in a specific lesion. Some success has already been seen when utilizing this technique in combination therapies, including one instance where photothermal ablation was performed in conjunction with multiple immunotherapies [30–32]; however, due to the broad differences that exist among the many variations of photothermal nanoparticles, we believe this topic deserves its own review.

## Immunotherapies

As a parallel to thermal ablation, many oncologists treat malignancies with immune modulating pharmaceuticals. As with ablation, the mechanisms of each immunotherapy varies greatly. However, these therapies can largely be divided into two categories; those that target the innate immune system, and those that target the adaptive. The innate immune system utilizes broad pattern recognition to respond rapidly, whereas adaptive responses are more specialized and can form the basis of long term immunologic memory [33]. While these two response types do differ greatly, they are both part of the larger immune response. Immunotherapies chiefly target either the innate or the adaptive immune responses, but methods that exploit the cross-talk between these two systems often exert the most robust effects.

Checkpoint inhibitors have seen a recent burst in clinical use as immunotherapies that target the adaptive immune response. There are several regulatory “checkpoints” that normally prevent the inappropriate activation of a cell-mediated immune response. By inhibiting these checkpoints, the immune system is freed to respond more robustly. Specifically, therapies exist that target and inhibit specific regulatory receptors. Cytotoxic T lymphocyte-associated antigen 4 (CTLA-4) binds costimulatory B7 molecules (CD80/86) with a much higher affinity than CD28. When B7 binds CTLA-4 instead of CD28, it does not produce its usual stimulatory signal. Therefore, CTLA-4 functions to competitively inhibit T cell stimulation, and promote T cell anergy [34]. Ipilimumab and Tremelimumab are both examples of anti-CTLA-4 antibodies that have demonstrated success at overcoming this regulatory hurdle. Ipilumimab is currently approved to treat metastatic melanoma while Tremelimuab is currently undergoing additional research [14, 35]. Similarly, programmed death receptor 1 (PD-1) is another inhibitory receptor found on T cells. When PD-1 is activated by PD-L1, a ligand often found on tumor cells, it inhibits T cell function and triggers apoptosis [34]. Pembrolizumab, Nivolumab, Durvalumab, and Avelumab are all anti-PD-1 drugs that have shown promise. The PD-1 inhibitors are approved for the treatment of melanoma, renal cell carcinoma, bladder cancer, non-small cell lung cancer, Hodgkins lymphoma, Merkel cell carcinoma and solid tumors. Both the CTLA-4 and PD-1 pathway have been implicated in the ability of tumors to evade the host immune system [34]. As such, both are prime targets for immunomodulation and for combining with ablation (Fig. 2). Another, somewhat more direct method for overcoming immune regulation is to directly target CD25+ FoxP3+ Regulatory T cells for depletion. Regulatory T cells are a subset function to suppress the immune system, and by administering anti-CD25 antibodies this inhibitory mechanism can be diminished [14, 36]. Varying extents of CD25+ Regulatory T cell depletion have also been shown following administration of the aforementioned CTLA-4 inhibitors [37, 38]. It should be noted that any therapy which depletes regulatory T cells via CD25+ is likely to also deplete some active, effector T cells which may transiently express CD25 [39].

Dendritic cells have been a major topic of investigation as possible targets for immunotherapy in the innate immune system. Dendritic Cells are professional antigen presenting cells and first line defenders that are able to pick up, process and present tumor antigens. Once activated by an infection, dendritic cells increase their expression of co-stimulators and can then go on to activate a larger lymphocyte response [14]. As one example the topical Toll-like Receptor 7 (TLR7) agonist Imiquimod has also been used to stimulate immature dendritic cells into inducing surface co-stimulators such as CD-80 and CD-86. Dendritic cells activated by Imiquimod go on to trigger type-1 helper T cell immunity [15]. As another example of innate immunomodulation, synthetic, repetitive bacterial nucleotides called CpG-oligodeoxynucleotides (CpG ODN) have been made to resemble specific unmethylated CpG dinucleotides from bacterial DNA. The Toll-Like receptor 9 is specific to these “CpG motifs” and thus CpG ODN are TLR-9 agonists. Professional antigen presenting cells such as dendritic cells and B cells utilize these TLR-9 receptors to induce an innate immune response to bacterial DNA [40, 41]. When CpG activates dendritic cells via TLR-9, they secrete IFN-α that triggers migration and clumping of more dendritic cells [42]. CpG activated TLRs also stimulate B cells to increase expression of co-stimulators, resist programmed cell death, upregulate the chemokine CCR7, and secrete Th-1 inducing mediators [41, 43]. Artificial CpG ODNs take advantage of this to stimulate an innate immune response. Critically, the route of administration of CpG B after local destructive therapy has been shown to affect its efficacy in mice. It has been shown that peritumoral administration confers increased rates of dendritic cell activation as well as an improved tumor specific CD8+ T cell response and protection against rechallenge as compared to both intravenous and distal cutaneous administration (*p* < 0.05) [44].

## Combination with innate immunotherapies

The addition of CpG B to radiofrequency ablation in a VX-2 rabbit model of liver cancer significantly increased mean survival, cytolytic activity, and tumor-specific T cell activation compared to either therapy alone. Additionally, the combined therapy demonstrated increased protection against pulmonary metastases when subjected to a rechallenge of injected malignant cells. Animals treated with combination RFA/CpG survived longer on average than those treated with RFA or CpG alone. Additionally, significantly less animals in the combination therapy group showed residual malignant tissue after 120 days compared to both monotherapy groups (*p* < 0.05). Combination therapy also produced the largest number of activated tumor specific lymphocytes compared to RFA alone, CpG alone, and the untreated controls as measured by a stimulation index (SI) (p < 0.05). A luciferase assay quantified the cytolytic activity of the activated T cells by assessing the levels of adenylate cyclase released by isolated peripheral T cells with relative luminescence units (RLU). Again, combination therapy produced the best outcome, followed by RFA alone, and CpG alone. There was no lytic activity in the untreated animals [40].

The addition of CpG B to cryoablative therapy has also been studied. Using a B16OVA mouse model of melanoma, this combination was shown to enhance dendritic cell maturation and cross presentation leading to a so-called “in-vivo dendritic cell vaccine.” While CpG alone had no effect on the growth of primary tumors or rechallenge, combination therapy completely protected against rechallenge with B16OVA. Interestingly, combination therapy also partially protected against a rechallenge after 40 days with wild-type, poorly immunogenic B16F10 tumors. Compared to untreated controls and monotherapy, combination therapy significantly increased CD80 expression on dendritic cells as well as MHC I and II presentation but did not significantly affect dendritic cell loading as compared to RFA monotherapy (*p* < 0.05). The combination of CpG B and cryoablation in this model also induced regression of existing secondary tumors in 40% of treated mice and completely protected against the outgrowth of local recurrences within 15 days after treatment [16].

Imiquimod, a TLR7 agonist and dendritic cell stimulator has also been combined with cryotherapy in a B16OVA murine model of melanoma. The combined administration of topical Imiquimod with cryoablation conferred significant protection against rechallenge. Combination therapy protected against rechallenge in 90% of the cases, compared to cryotherapy alone which only protected 30% of cases (*p* < 0.0001). The combination also increased T cell proliferation as well as IFN-γ production when compared to either therapy alone. IFN-γ was measured in-vitro after stimulation with OVA protein. Cryotherapy plus Imiquimod induced more than double the production of IFN-γ compared to cryotherapy alone [15].

In addition to stimulating in-vivo dendritic cells, much research has been done to investigate the benefits of combining ablative therapy with an injection of ex-vivo dendritic cells directly into the tumor site. Ablative therapy in combination with an injection of either immature or stimulated dendritic cells has been studied. One study used a MB49 murine model of urothethial carcinoma to show that RFA and injection of immature ex vivo dendritic cells each independently result in enhanced antitumor T cell response and tumor regression, but that the combination of the two does not amplify this response [45]. In contrast, another study showed that intratumoral injection of immature dendritic cells alone increased the proliferation rate of CD8+ T cells, but only the combined administration of both immature dendritic cells and cryoablation generated effector memory cells. They also showed that the combination of ex-vivo immature dendritic cells and cryotherapy conferred significantly prolonged survival following amputation of the foot bearing the primary tumor and after rechallenge (*p* = 0.005 and *p* = 0.029 respectively) [46].

Nakagawa et al. demonstrated that administration of dendritic cells stimulated by OK-432 (an antigen derived from the Su strain of group A Streptococcus pyogenes) after RFA conferred a significant decrease in mean tumor volume compared to RFA alone or RFA with administration of immature dendritic cells (*p* < 0.001). Additionally they showed that combination therapy significantly increased the number of CD8+ T Cells infiltrating untreated secondary tumors as compared to RFA alone or RFA with immature dendritic cells (p < 0.001) [47]. Another study compared the administration of ex-vivo, heat-shocked tumor cell lysate-pulsed dendritic cells (HT-DC) to the administration of ex-vivo, unheated tumor lysate pulsed dendritic cells prior to treatment with RFA. The addition of HT-DC’s significantly prolonged survival and increased the IFN-γ produced by CD8+ T cells compared to combination with unheated tumor lysate-pulsed dendritic cells (*p* < 0.01). Additionally, they also proved that transfer of splenocytes from animals successfully treated with RFA and HT-DC to naïve ones conferred protection from recurrence [48].

Radiofrequency ablation has also been combined with a poxviral vaccine expressing carcinoembryonic antigen (CEA) and a triad of costimulatory molecules (TRICOM). This combination was shown to induce regression of distal metastatic tumors when either therapy alone did not. Additionally, adding the vaccine to sequential RFA significantly reduced the combined volume of primary and distal tumors (*p* < 0.0054). Combination therapy also prolonged relapse-free survival as compared to RFA monotherapy. Combination therapy eliminated 100% of primary tumors, compared to 43% by RFA alone. The level of CEA specific CD4+ response was also highest in the combination group compared to either monotherapy (*p* < 0.0003). This combined therapy of CEA/TRICOM vaccine and RFA also greatly increased the transcription of two tumor-suppressor micro RNAs, miR-141 and miR-205, as compared to either therapy alone. The tumor suppressing micro RNA, miR-150 was increased by all three therapies, and miR-133b was decreased by the combined therapy [49].

Administration of an active variant of CC chemokine ligand 3, ECI301, after radiofrequency ablation significantly reduced volume of tumors and significantly increased CD4+ and CD8+ T cell infiltration in a murine model of hepatocellular carcinoma (*p* < 0.05 & *p* < 0.01). These effects were eliminated CCR1-deficient mice, but not in CCR-5 deficient mice, indicating the effect is mediated by CCR1 [50].

Li, et al. used microwave ablation in combination with OK-432, a penicillin inactivated and lyophilized preparation of a low virulence strain of group A streptococcus pyogenes, as an immunostimulant to induce the innate immune system to produce inflammatory cytokines in a murine model of breast cancer. Compared to controls, combination therapy significantly prolonged survival after ablation and significantly decreased the volume of tumors arising in animals after rechallenge (*p* < 0.001 & *p* < 0.05). After the rechallenge, the majority of animals treated with combination therapy completely rejected secondary tumors, whereas none of the control mice did. Combination therapy also significantly increased the infiltration of CD8+ T cells into tumors compared to ablation monotherapy, but did not significantly increase the infiltration of CD4+ T cells. Combination therapy also significantly increased the percentage of splenic CD4+ and CD8+ T cells compared to monotherapy (*p* < 0.05 & *p* < 0.01). They also characterized the ratio of Th1 (IFN-γ) to Th2 (IL-4) cytokines produced by CD4+ T cells in the different treatment groups. Combination therapy had a higher percentage of IFN-γ producing cells compared to monotherapy and a lower percentage of IL-4 producing cells compared to controls (*p* = 0.004 & *p* = 0.05). Overall the ratio of Th1 to Th2 was significantly higher in compared to the controls with combination therapy, but not with ablation alone (*p* < 0.05). Specific Th1 cytokines IL-18, IL-2, and IL-12 were all shown to be significantly increased with combination therapy compared to ablation alone (*p* < 0.01, *p* < 0.05, and *p* < 0.01) [51].

## Combination with adaptive immunotherapies

Several immunotherapies that target the adaptive system have also been investigated. Den Brok, et al. showed that inhibition of CTLA-4 with specific antibodies prior to either RFA or cryoablation amplifies the response to treatment [14, 52]. The combination of either cryoablation or RFA with CTLA-4 inhibition in a B16OVA model of melanoma conferred a significant increase in survival after rechallenge as compared to untreated controls and combination with sham IgG antibodies (*p* < 0.05) [14]. The authors additionally demonstrated an increase in tumor specific T cells 10 days after both cryotherapy and RFA combination treatment by identifying OVA kb tetramer positive CD8b + T cells. Following both RFA/anti-CTLA-4 and Cryoablation/anti-CTLA-4 therapy the percentage of OVA+ CD8b + T cells also increased, whereas the percentage did not increase in controls. It is notable that the loading of tumor antigens onto in vivo dendritic cells was more efficient with cryoablation than RFA, as measured by using CD11+ beads to sort out dendritic cells [14]. Additional research has shown that adding the CTLA-4 inhibitor Ipilumimab to cryoablation therapy confers an improved response in a mouse model of prostate cancer. When compared to either therapy alone, combination therapy significantly increased the infiltration of CD4+ and CD8+ T cells into tumors and elevated the ratio of effector CD4+ cells to regulatory FoxP3+ T cells as compared to monotherapy (*p* < 0.01–0.05). Additionally, the authors showed that combination therapy significantly prolonged tumor free survival (*p* < 0.0005). Finally, they demonstrated that following a challenge with a new secondary tumor 1 day after treatment, none of the mice receiving cryotherapy alone rejected the tumor whereas almost half of the mice receiving combination therapy did [35].

Den Brok et al. also demonstrated the effectiveness of combining ablation with anti-CD25 antibodies. Both cryoablation and RFA in combination with anti-CD25 antibodies showed a significant increase in survival following rechallenge compared (*p* < 005). The percentage of OVA kb tetramer positive CD8b + T cells 10 days after treatment was also quantified. Following both RFA/anti-CD25 and Cryoablation/anti-CD25 therapy, the percentage increased, but it did not increase following monotherapy [14]. Cyclophosphamide has also been used to deplete regulatory T cells prior to cryoablation of a solid malignancy. When Cyclophosphamide was combined with cryoablation in a murine colorectal model, the proliferation of tumor specific T cells was greatly increased as was the ratio of effector CD4+ T cells to regulatory FoxP3+ T cells. Survival in animals that received combination therapy was significantly prolonged and the combination also significantly increased protection against rechallenge with malignant cells after 150 days of survival (*p* < 0.0001 & *p* = 0.0051). This increased antitumor immunity was successfully transferred to naïve animals by injecting them with lymphocytes from tumor draining lymph nodes of the treated animals. The transfer of immunity was dependent on the presence of CD8+ T cells from treated animals. Removal of CD4+ T cells from the infusate had no effect on the conferred immunity. This indicates that CD8+ T cells are the main effector of antitumor immunity [53].

Another study found that addition of anti-PD-1 antibodies to RFA in a murine colon cancer model overcomes a major checkpoint to a systemic immune response. They found that tumors significantly upregulated the regulatory co-stimulators PD-1 in response to RFA. By adding PD-1 inhibition to RFA, they achieved significant a decrease in tumor volume and a significant increase in survival (*p* < 0.001) [54].

Chen et al. conducted a unique study that compared the effects of an innate immunotherapy plus microwave ablation to a combination of innate and adaptive immunotherapies plus microwave ablation. First off, they combined an intratumoral injection of microspheres encapsulating granulocyte-macrophage colony stimulating factor (GM-CSF) with microwave ablation in a murine model of hepatoma. They chose GM-CSF microspheres because GM-CSF is known to be highly efficacious at recruiting and activating dendritic cells. The albumin based spheres released GM-CSF over 3 days, and 3 separate injections were given. Bovine Serum Albumin Microspheres (sham-BSA) were used a control. Following a rechallenge with malignant cells 8 weeks after treatment, microwave ablation combined with GM-CSF was shown to significantly increase the percent of animals surviving tumor-free and to significantly decrease the tumor volume (*p* < 0.01 & *p* = 0.0183). The response to treatment was even more profound with the addition of anti-CTLA-4 antibodies for a combined MWA/GM-CSF/anti-CTLA-4 therapy. This 3 therapy combination significantly increased total survival after initial inoculation compared to untreated animals (*p* < 0.002). Additionally, after rechallenge the combination significantly increased the percentage of mice surviving tumor-free and significantly decreased the volume of tumors in mice 6–7 weeks after rechallenge (*p* = 0.0189 & *p* < 0.02). Furthermore the triple therapy protected against rechallenge in almost all of the mice treated, and cured distal tumors in half of the mice with small tumor burden [55] (Tables 2, 3 and 4).

**Table 2:** Studies that investigated the effects of combining radiofrequency ablation with immunomodulation and their results

Radiofrequency Ablation and Immunotherapy					
Reference	Tumor Model	Immunotherapy	Endpoints	RFA Monotherapy	Combination Therapy
Behm, B., et al. [31]	VX2 Rabbit Liver Cancer	CpG B	Mean Animal Survival After Inoculation	97.3 Days (Range: 76–120) (*p* < 0.05 versus Control)	113.9 Days (Range: 89–120) (*p* < 0.05 versus Control & Monotherapy)
			Tumor Specific Lymphocyte Stimulation Index 2 Weeks After Treatment	88.49 SI (39.30 StdDev) (*p* < 0.05 versus Control)	103.43 SI (55.55 StdDev) (*p* < 0.05 versus Control)
			Cytolytic Activity 2 Weeks After Treatment^NS^	487.79 RLU (148.69 StdDev) (Not Significant versus Control)	972.72 RLU (362.77 StdDev) (*p* < 0.05 versus Control)
			Rechallenge With Malignant Cells to Animals Surviving 160 Days	All Animals Developed Pulmonary Metastases	Pulmonary Metastases Developed in 2 out of 6 Animals
den Brok, M.H., et al. [10]	B16OVA Murine Melanoma	Anti-CTLA-4 Antibodies	OVA Rechallenge to Animals Surviving 40 Days	Used as Control with Sham IgG Antibodies	Significant Increase in Percentage Survival Versus Control (*p* < 0.05)
			OVA kb Tetramer Positive CD8b + T Cells After 10 Days	0.1% of CD8b + T Cells	4.2% of CD8b + T Cells
		Anti-CD25 Antibodies	OVA Rechallenge to Animals Surviving 40 Days	Used as Control with Sham IgG Antibodies	Significant Increase in Percentage Survival Versus Control (*p* < 0.05)
			OVA kb Tetramer Positive CD8b + T Cells After 10 Days	0.1% of CD8b + T Cells	2.8% of CD8b + T Cells
Dromi, S.A., et al. [36]	MB49 Murine Urothethial Carcinoma	Intratumoral Injection of Immature Ex-Vivo Dendritic Cells	Median Tumor Volume Change	Significant Volume Loss Versus Control (*p* < 0.05 versus Control)	No Significance Established Versus Control or Monotherapy (*p* = 0.07)
Nakagawa, H., et al. [38]	MC38 Murine Colon Cancer	Local Injection of OK-432 Stimulated Dendritic Cells	Mean Tumor Volume 10 Days After Treatment	No Significant Established Versus Control	Significant Volume Loss Versus Monotherapy (*p* < 0.001)
			CD4+ T Cell Infiltration into Untreated Tumors 10 Days After Treatment	Significantly Increased Versus Control (*p* < 0.001)	Significantly Increased Versus Control & Monotherapy (*p* < 0.001)
			CD8+ T Cell Infiltration into Untreated Tumors 10 Days After Treatment	Significantly Increased Versus Control (*p* < 0.001)	Significantly Increased Versus Control & Monotherapy (*p* < 0.001)
Liu, Q., et al. [39]	B16F10-luc Murine Melanoma	Heat-shocked Tumor Cell Lysate-pulsed Dendritic Cells	Percent Survival 100 Days After Treatment	No Data	Significantly Increased Versus Combination with Unheated Tumor Cell Lysate Pulsed DC’s (*p* < 0.01)
			IFN-γ Produced by CD+ T Cells Isolated From Draining Lymph Nodes After 2 Weeks	No Data	Significantly Increased Versus Combination with Unheated Tumor Cell Lysate Pulsed DC’s (*p* < 0.01)
Gameiro, S.R., et al. [40]	MC38 CEA+ Murine Colon Cancer	Poxviral Vaccine Expressing CEA and a TRIad of Costimulatory Molecules (CEA/TRICOM)	Primary & Distal Tumor Burden at Day 24	Combined Volume Significantly Reduced Versus Control (*p* < 0.0001)	Combined Volume Significantly Reduced Versus Monotherapy (*p* < 0.0054)
			Relapse Free Survival	34.5% Animals Achieved Tumor Eradication 50% Subsequently Relapsed	52% Animals Achieved Tumor Eradication 30.8% Subsequently Relapsed
Iida, N., et al., [41]	BNL 1ME A.7R.1 Murine Hepatoma	Active Variant of CC Chemokine Ligand 3 (ECI301)	Tumor Volume After 14 Days	Significantly Reduced Compared to Control (*p* < 0.01)	Significantly Reduced Versus Monotherapy (*p* < 0.05)
			CD4+ & CD8+ T Cell Infiltration After 3 Days	Significantly Increased Versus Control (*p* < 0.01)	Significantly Increased Versus Monotherapy (*p* < 0.01)
Shi, L., et al. [16]	CT26 Murine Colon Cancer	Anti-PD-1 Antibodies	Tumor Volume After 30 Days	No Significance Established Versus Control	Significantly Reduced Versus Monotherapy (*p* < 0.001)
			Total Survival After Inoculation	No Significance Established Versus Control	Significantly Prolonged Survival Versus Monotherapy (*p* < 0.001)

Cytolytic activity as measured by relative luminescence units (RLU) of adenylate kinase released by isolated peripheral blood T cells

NS, not significant

**Table 3:** Studies that investigated the effects of combining cryoablation with immunomodulation and their results

Cryoablation and Immunotherapy					
Reference	Tumor Model	Immunotherapy	Endpoint	Ablation Monotherapy	Combination Therapy
den Brok, M.H., et al. [12]	B16OVA Murine Melanoma	CpG	Total Number of Dendritic Cells per Lymph Node 2 Days After Treatment	No Significance Established Versus Control	Significantly Increased Versus Monotherapy (*p* < 0.05)
			CD80 Expression on OVA Specific Dendritic Cells 2 Days After Treatment	Significantly Increased Versus Control (*p* < 0.05)	Significantly Increased Compared to Monotherapy (*p* < 0.05)
			MHC Class I Presentation (B3Z Activation)	No Significance Established Versus Control	Significantly Increased Versus Monotherapy (*p* < 0.05)
			MHC Class II Presentation (IL-2 Production)	Significantly Increased Versus Control (*p* < 0.05)	Significantly Increased Versus Monotherapy (*p* < 0.05)
			OVA Rechallenge to Animals Surviving 40 Days	Significantly Increased Survival Versus Control (*p* < 0.005)	Complete Protection Against Tumor Outgrowth (*p* < 0.005)
			Survival From Contralateral Metastasis	No Significance Established Versus Control	Significantly Prolonged Versus Monotherapy (*p* < 0.005)
			Local Recurrence Within 15 Days	Significantly Higher Recurrence Versus Combination Therapy (*p* < 0.05)	Complete Protection Against Outgrowth of Local Recurrences
Machlenkin, A. [37]	3LL Murine Lewis Lung Carcinoma & B16OVA Murine Melanoma	Intratumoral Injection of Immature Dendritic Cells	Survival Following Amputation of Foot Bearing Primary Tumor	No Significance Established Versus Control	Significantly Prolonged Versus Monotherapy (*p* = 0.005)
			Proliferation of Tumor Specific CD8+ T Cells	35% of CD8+ T Cells Underwent Division	65% of CD8+ T Cells Underwent Division
			Rechallenge With Malignant Cells to Animals Surviving 60 Days	No Significance Established Versus Control	Significantly Prolonged Survival Versus Monotherapy (*p* = 0.029)
Redondo, P., et al. [11]	B16OVA Murine Melanoma	Topical TLR-7 Agonist Imiquimod	Rechallenge With Malignant Cells to Animals Surviving 15 Days	Significantly Delayed the Outgrowth of Secondary Tumors Compared to Control (*p* < 0.0001)	Significantly Protection Against Secondary Tumor Outgrowth Compared to Monotherapy (*p* < 0.0001)
Waitz, R., et al. [15]	TRAMP C2 Murine Prostate Cancer	Ipilumimab CTLA-4 Inhibition	Tumor Free Survival	No Significance Established Versus Control	Significantly Prolonged Versus Monotherapy (*p* < 0.0005)
			Challenge With Secondary Tumor After 1 Day	0 out of 5 Mice Rejected the Secondary Tumor	4 out of 9 Mice Rejected the Secondary Tumor
			Number of Infiltrating CD4+ T Cells After 15 Days	No Significance Established Versus Control	Significantly Increased Versus Control Only (*p* = 0.01–0.05)
			Number of Infiltrating CD8+ T Cells After 15 Days	No Significance Established Versus Control	Significantly Increased Versus Monotherapy (*p* < 0.001)
			Ratio of Intratumoral CD4+ Effector T cells to FoxP3 Regulatory T cells	No Significance Established Versus Control	Significantly Increased Versus Monotherapy (*p* < 0.001)
			Ratio of Intratumoral CD8+ Effector T cells to FoxP3 Regulatory T cells	No Significance Established Versus Control	Significantly Increased Versus Monotherapy (*p* = 0.001)
Levy, M.Y., et al. [44]	CT26 Murine Colon Cancer	Cyclophosphamide	Total Survival After Inoculation	Significance Not Established Versus Control (*p* = 0.46)	Significantly Prolonged Versus Monotherapy (*p* < 0.0001)
			Rechallenge With Malignant Cells to Animals Surviving 150 Days	No Significance Established Versus Control	Significantly Prolonged Survival Versus Controls (*p* = 0.0051)
den Brok, M.H., et al. [10]	B16OVA Murine Melanoma	Anti-CTLA-4 Antibodies	OVA Rechallenge to Animals Surviving 40 Days	Used as Control with Sham IgG Antibodies	Significant Increase in Percentage Survival Versus Control (*p* < 0.05)
			OVA kb Tetramer Positive CD8b + T Cells After 10 Days	0.1% of CD8b + T Cells	5.8% of CD8b + T Cells
		Anti-CD25 Antibodies	OVA Rechallenge to Animals Surviving 40 Days	Used as Control with Sham IgG Antibodies	Significant Increase in Percentage Survival Versus Control (*p* < 0.05)
			OVA kb Tetramer Positive CD8b + T Cells After 10 Days	0.1% of CD8b + T Cells	5.0% of CD8b + T Cells

**Table 4:** Studies that investigated the effects of combining microwave ablation with immunomodulation and their results

Microwave Ablation and Immunotherapy					
Reference	Tumor Model	Immunotherapy	Endpoint	Ablation Monotherapy	Combination Therapy
Li, L., et al. [42]	4 T1 Murine Breast Cancer	OK-432	Total Survival After Treatment	No Significance Established Versus Control	Significantly Prolonged Compared to Monotherapy (*p* < 0.001)
			Rechallenge to Surviving Mice 25 Days After Treatment	No Data	Significantly Decreased Tumor Volume Versus Control After 25 Days (*p* < 0.05)
			Infiltration of CD4+ T Cells Into Tumors	Significantly Increased Versus Control (*p* < 0.001)	Significantly Increased Versus Control NOT Monotherapy (*p* < 0.001)
			Infiltration of CD8+ T Cells Into Tumors	Significantly Increased Versus Control (*p* < 0.001)	Significantly Increased Versus Monotherapy (*p* < 0.001)
			Percentage of Splenic CD4+ T Cells	Significantly Increased Versus Control (*p* < 0.01)	Significantly Increased Versus Monotherapy (*p* < 0.05)
			Percentage of Splenic CD8+ T Cells	Significantly Increased Versus Control (*p* < 0.001)	Significantly Increased Versus Monotherapy (*p* < 0.01)
			Number of 4 T1 Specific IFN-γ Secreting Cells	Significantly Increased Versus Control (*p* < 0.001)	Significantly Increased Versus Monotherapy (*p* = 0.031)
			Ratio of Th1 to Th2 Cytokines Produced by CD4+ T Cells	No Significance Established Versus Control	Significantly Increased Versus Monotherapy (*p* < 0.05)
			Levels of Plasma IL-18 7 Days After Treatment	No Significance Established Versus Control	Significantly Increased Versus Monotherapy (*p* < 0.01)
			Levels of Plasma IL-2 7 Days After Treatment	No Significance Established Versus Control	Significantly Increased Versus Monotherapy (*p* < 0.05)
			Levels of Plasma IL-12 7 Days After Treatment	Significantly Increased Versus Control (*p* < 005)	Significantly Increased Versus Monotherapy (*p* < 0.01)
Chen, Z., et al. [45]	Hepa 1–6 Murine Hepatoma	Intratumoral Microspheres Encapsulating GM-CSF	Tumor Free Survival Following Rechallenge to Animals Surviving 8 Weeks	Used as Control with Sham BSA Microspheres	Significant Increase in Percent Tumor Free Survival Versus Monotherapy After 6–7 Weeks (*p* < 0.01)
			Tumor Volume Following Rechallenge to Animals Surviving 8 Weeks	Used as Control with Sham BSA Microspheres	Significant Decrease Versus Monotherapy After 8 Weeks (*p* = 0.0183)
		GM-CSF Microspheres + Anti-CTLA-4 Antibodies	Tumor Free Survival Following Rechallenge to Animals Surviving 8 Weeks	MWA/GM-CSF/Sham IgG Antibodies Used as Control	Significant Increase in Percent Tumor Free Survival Versus Control After 6–7 Weeks (*p* = 0.0189)
			Tumor Volume Following Rechallenge to Animals Surviving 8 Weeks	MWA/GM-CSF/Sham IgG Antibodies Used as Control	Significant Decrease in Tumor Volume Versus Control After 8 Weeks (*p* < 0.02)
			Total Survival After Inoculation	MWA/GM-CSF/Sham IgG Antibodies Used as Control	Significantly Prolonged Compared to Control (*p* < 0.002)

## Conclusion

The benefits of combining immunotherapy with ablation continue to be unraveled. In particular, much work still needs to be done to elucidate the effects induced through the combination of ablation with immunotherapies that target the adaptive immune system. Regardless, the synergistic enhancement of the anticancer immune response exhibited by the combination therapies in these early studies shows great promise for the future of oncologic treatment.

## Abbreviations

ATP: Adenosine triphosphate
BSA: Bovine serum albumin
CD: Cluster of differentiation
CEA: Carcinoembryonic antigen
CpG ODN: CpG-oligodeoxynucleotides
CTLA-4: Cytotoxic T lymphocyte-associated antigen 4
DAMPs: Damage associated molecular patterns
DCs: Dendritic cells
FoxP3: Forkhead box P3
FUS: Focused ultrasound
GM-CSF: Granulocyte-macrophage colony stimulating factor
HIFU: High Intensity Focused ultra sound
HMGB1: High mobility group protein B1
HSPs: Heat shock proteins
HT-DC: Heat-Shocked Tumor Cell lysate-pulsed dendritic cells
IFN-γ: Interferon γ
LOFU: Low energy focused ultrasound
MHC: Major histocompatability complex
MWA: Microwave ablation
NF-κβ: Nuclear factor kappa-light-chain-enhancer of activated B cells
PD: Programmed death receptor
RFA: Radiofrequency ablation
RLU: Relative luminescence units
SABR: Stereotactic ablative radiotherapy
SBRT: Stereotactic body radiation therapy
SI: Stimulation index
TGF-β1/2: Transforming growth factor beta 1/2
TLR: Toll-like receptor
TNF-α: Tumor necrosis factor alpha
TRICOM: Triad of costimulatory molecules
VEGF: Vascular endothelial growth factor
Y90: Yttrium 90
